# A randomized controlled trial on the efficacy and safety of vitamin A supplementation in children with sepsis

**DOI:** 10.3389/fped.2025.1579006

**Published:** 2025-08-06

**Authors:** Qinyi Fu, Maoxia Liu, Xuepeng Zhang, Jianlei Fu, Geng Zhang, Yi Ji, Siyuan Chen

**Affiliations:** ^1^ Department of Critical Care Medicine, West China Hospital of Sichuan University, Chengdu, China; ^2^ Division of Oncology, Department of Pediatric Surgery, West China Hospital of Sichuan University, Chengdu, China

**Keywords:** sepsis, vitamin A, PICU, lactate, albumin, inflammation

## Abstract

**Background:**

Our previous research confirmed that vitamin A (VA) deficiency commonly occurs in children with sepsis, and serum VA levels negatively correlate with disease severity. This study aimed to evaluate the efficacy and safety of VA supplementation (VAS) in children with sepsis.

**Methods:**

A randomized, single-blind, single-center trial was conducted from June 2020 to March 2024 involving children diagnosed with sepsis. Participants were randomly allocated to either the VAS group or the placebo group. The primary outcome was length of stay in the ICU, and secondary outcomes included hospital length of stay, 28-day mortality, duration of mechanical ventilation, and antibiotic usage. Vital signs, clinical symptoms, and laboratory data were recorded, and statistical analyses assessed the efficacy and safety of VAS.

**Results:**

A total of 156 children with sepsis were enrolled: 72 in the VAS group and 84 in the placebo group. The median baseline VA level among participants was 147.22 (97.20–258.04) ng/ml. There was no significant difference in ICU length of stay between the VAS and placebo groups [14 (7–27) vs. 16.5 (9.3–26) days, *P* = 0.451]. Similarly, no significant differences were observed between groups regarding hospital length of stay, 28-day mortality, duration of mechanical ventilation, or antibiotic usage. In addition, we performed a *post hoc* analysis of biomarkers. VAS showed a significant downward trend in lactate levels (*P* < 0.001), higher 24-h lactate clearance rate (25%), and significantly lower lactate levels on the third day post-intervention (1.28 ± 0.50 vs. 2.20 ± 2.21, *P* = 0.001). Additionally, VA levels positively correlated with serum albumin (ALB) (*r* = 0.479, *P* < 0.001). Procalcitonin (PCT) levels were significantly lower in the VAS group on the 3rd (*P* = 0.032) and 7th day (*P* = 0.001) post-intervention, and white blood cell (WBC) count was lower on the 3rd day (*P* = 0.022). Furthermore, compared with the placebo group, the VAS group had a greater reduction in PCT levels within 24 h following intervention (73% vs. 48%, *P* = 0.001). No adverse reactions associated with VAS were observed.

**Conclusions:**

VAS did not significantly reduce ICU length of stay or 28-day mortality in children with septic shock, however, it may have beneficial effects on systemic inflammation and lactate metabolism. Further studies are warranted to explore the relationship between VA and ALB.

**Clinical Trial Registration:**

Clinicaltrials.gov, identifier NCT04127968.

## Introduction

Sepsis is a life-threatening organ dysfunction caused by an immune dysregulated host response to infection. It is characterized by a complex syndrome involving protective immune abnormalities (1) and represents a common cause of mortality among critically ill children, with reported mortality rates as high as 34.6% (2). Over recent decades, the incidence of sepsis has steadily increased (3), posing a significant burden on healthcare resources and becoming an important public health concern. Currently, no specific treatment for sepsis is available; management primarily relies on anti-infective measures and supportive therapies aimed at organ protection. Identifying effective adjunctive interventions to improve prognoses in pediatric sepsis has therefore become an urgent priority.

VA and its derivatives constitute a group of unsaturated fat-soluble organic compounds essential for visual regulation, infection resistance, maintenance of epithelial integrity, tissue growth, metabolism, and reproductive functions. Although these compounds exist in trace amounts, even minor fluctuations in their concentrations can lead to substantial physiological and pathological effects. For instance, abnormal tissue concentrations of retinoic acid (RA), a derivative of VA, can lead to adverse outcomes, including congenital defects, immune deficiencies, abnormal cell proliferation, and toxicity (4). Beyond the well-established link between VA deficiency (VAD) and night blindness, extensive literature suggests that VAD is associated with poor health outcomes due to increased susceptibility to infections in children. VAD may impair immunity through multiple mechanisms, including disruption of gastrointestinal mucosal barrier integrity, increased respiratory epithelial damage with impaired repair processes, decreased numbers of monocytes and natural killer (NK) cells, and compromised functions of macrophages, dendritic cells, and neutrophils (5–8). Our previous research indicated that VAD is associated with decreased interferon-alpha (IFN-α) and enterovirus 71 (EV71)-specific immunoglobulin M (IgM) levels, thereby compromising immune function and increasing disease severity in children with EV71 infection (9, 10). Subsequently, we identified widespread low serum VA levels in children with sepsis, showing that VAD correlated significantly with severe sepsis, septic shock, and elevated Pediatric Risk of Mortality III (PRISM III) scores (11, 12). However, whether VAS can improve clinical outcomes in pediatric sepsis remains unclear and warrants further investigation.

According to reports, adverse reactions associated with a single high-dose administration of VA include rash, transient cerebral edema accompanied by vomiting, and symptoms such as headache, drowsiness, nausea, and vomiting in certain cases (13). Chronic toxicity can result in skin, hair, and nail changes; abnormal liver function test results; and fetal birth defects. Both acute and chronic forms can lead to headaches and intracranial hypertension (14). Unless birth defects have occurred, dose adjustments typically lead to full recovery (15). Therefore, it is essential to monitor side effects related to VAS and evaluate the safety of VA interventions.

In this study, we conducted a randomized controlled trial and analyzed relevant indicators in both the VAS group and the placebo group. Our aim was to investigate the efficacy and safety of VAS in children with sepsis and provide robust clinical evidence supporting adjunctive therapeutic strategies in pediatric sepsis management.

## Materials and methods

### Study design

This was a single-center, single-blind, randomized controlled trial. The study included children with sepsis from the Pediatric Intensive Care Unit (PICU) of the Department of Critical Care Medicine at West China Hospital, Sichuan University, from June 2020 to March 2024.

### Participants

**Figure 1:**
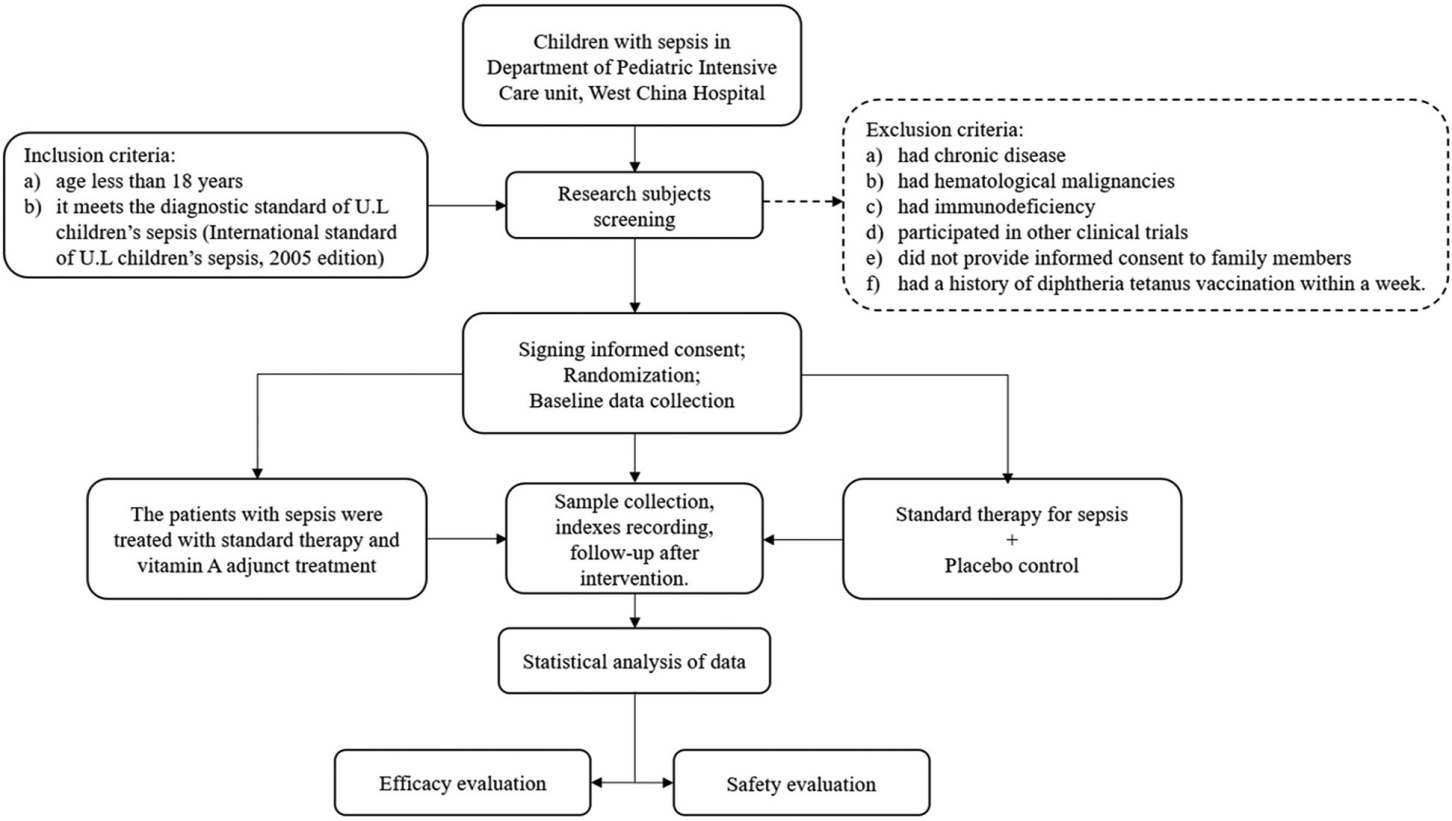
Flow chart of clinical research.

Children admitted to the PICU were eligible for inclusion if they (a) were younger than 18 years and (b) met the diagnostic criteria for pediatric sepsis (16). Patients were excluded if they (a) had chronic disease, (b) had hematological malignancies, (c) had immunodeficiency disorders, (d) participated in other clinical trials, (e) refused to provide consent, or (f) had a history of diphtheria-tetanus-pertussis (DTP) vaccination within 1 week prior to enrollment (Figure 1). [Limited data suggest an increased incidence of acute adverse effects when VAS is concurrently administered with DTP vaccination (17). Additionally, one study indicates higher mortality in children receiving both VA and DTP vaccine compared with children receiving VA alone or no intervention (18). To maximize clinical safety, participants who received DTP vaccination within the preceding week were excluded from this study].

### Sample size calculation

The primary endpoint of this trial was ICU length of stay, analyzed using a two-sample *t*-test. Preliminary pilot study data indicated that the mean ICU stay was 13.79 days in the experimental group and 16.96 days in the control group, with a standard deviation of 6.34 days. Assuming equal sample sizes in both groups, a significance level (α) of 0.05 (two-sided), and a statistical power (1-β) of 0.8, the required sample size per group was calculated using the Two-Sample *T*-Test Allowing Equal Variance option under the Means menu in PASS 21.0 software. The calculation indicated a necessary enrollment of 64 participants per group. Allowing for an anticipated dropout rate of 10%, a total of 72 participants per group (144 participants in total) were enrolled to ensure the accuracy and scientific validity of the study results.

### Intervention

Participants enrolled in this study were randomly assigned to either the VAS group or the placebo group. The administered intervention followed the World Health Organization's recommended safe VAS doses based on age group, as outlined in 2011 (Table 1) (19). The placebo group received an equivalent volume of saline. All enrolled patients received standardized enteral or parenteral nutrition according to PICU guidelines. Nutritional intake, including VA from dietary sources, was monitored and recorded daily but was not included in intervention dosing calculations. VA concentrations were measured at baseline and again on day 3 post-intervention. Parents and patients remained blinded to treatment allocation throughout the study. Serum VA levels below 200 ng/ml were defined as deficient (20). Serum VA levels were measured by trained personnel in the Department of Laboratory Medicine, West China Hospital of Sichuan University, using high-performance liquid chromatography (HPLC) with ultraviolet detection. The mobile phase consisted of solvent A (ddH_2_O containing 0.1% formic acid) and solvent B (100% acetonitrile), with rigorous quality control protocols maintained throughout the analytical process. According to International Consensus criteria (16), sepsis accompanied by acute respiratory distress syndrome, cardiovascular dysfunction, or dysfunction of two or more additional acute organ systems was defined as severe sepsis. Sepsis presenting with cardiovascular dysfunction was defined as septic shock. Organ dysfunction was classified in accordance with these international guidelines (16).

**Table 1:** Intervention dose of VA in children of different age groups (40).

Age group	VA dose (IU)
<6 months	5,000
6 months–12 months	10,000
12 months–12 years old	20,000

Patient characteristics including age, sex, weight, ethnicity, pediatric sequential organ failure assessment (pSOFA) scores, PRISM III scores, and infection site were recorded. Blood samples were collected at baseline and on days 1, 3, and 7 following VAS to perform laboratory tests, including blood gas analysis, routine blood counts, liver function tests, kidney function tests, PCT measurements, and absolute T-cell counts. ICU length of stay, hospital length of stay, and 28-day mortality rates were documented to evaluate the efficacy of VA intervention. Additionally, key treatment variables such as duration of mechanical ventilation, antibiotic use, incidence of hypoglycemia, and the administration of vasoactive drugs and blood products were recorded. Safety assessments involved continuous monitoring of patients' vital signs, clinical manifestations, and laboratory results.

### Outcomes

The primary outcome measure was the ICU length of stay among pediatric patients with sepsis. Secondary outcomes included total hospital length of stay and 28-day mortality. Additionally, as a *post hoc* analysis, we compared the duration of mechanical ventilation, antibiotic usage, incidence of severe sepsis/septic shock, occurrence of hypoglycemia, utilization of vasoactive medications and blood products, dynamic trends in lactate levels, and relevant laboratory parameters between the two groups. For patients who died or were discharged from the ICU before day 7, the last-observation-carried-forward method was employed. All primary infections, including pneumonia, bloodstream infections, intra-abdominal infections, skin and soft-tissue infections, intracranial infections, multiple infections, and other infections, were documented. Considering potential adverse effects associated with VAS, we monitored the incidence of headache, vomiting, irritability, blurred vision, delayed closure or fullness of the fontanelle, decreased appetite, weight loss or no weight gain, dry skin, scaly desquamation, itching, rash, cracked lips, dry and brittle hair, hair loss, and muscle pain. All unexpected serious adverse events (i.e., those neither prespecified nor included as outcomes) considered by the investigator to be possibly related to the trial procedure were reported to the trial coordinating center within 24 h.

### Statistical analysis

To control for potential confounding factors, sensitivity analyses were conducted using multivariable regression models adjusted for clinically relevant covariates. Variables included infection source (categorized as pulmonary, bloodstream, intra-abdominal, skin/soft tissue, intracranial, multiple infections, or other), surgical status (postoperative vs. nonsurgical), and baseline disease severity as assessed by PRISM III scores. Adjusted estimates are reported alongside unadjusted results. Statistical analyses were performed on an intention-to-treat basis using SPSS software (version 25.0). Quantitative variables are presented as the mean (standard deviation) or median (interquartile range). Normally distributed continuous variables were compared between the two groups using independent-samples *t*-tests, with results reported as mean ± standard deviation (SD). Nonnormally distributed continuous variables were analyzed using the Mann–Whitney *U* test, with results reported as median (interquartile range). Categorical variables were analyzed using the chi-square test or Fisher's exact test, with results expressed as counts (percentages). Generalized estimating equations were applied to analyze repeated lactate measurements at three time points. Correlation analyses were conducted using Pearson's or Spearman's correlation tests as appropriate. Statistical significance was set at *P* < 0.05.

## Results

### Patients

**Figure 2:**
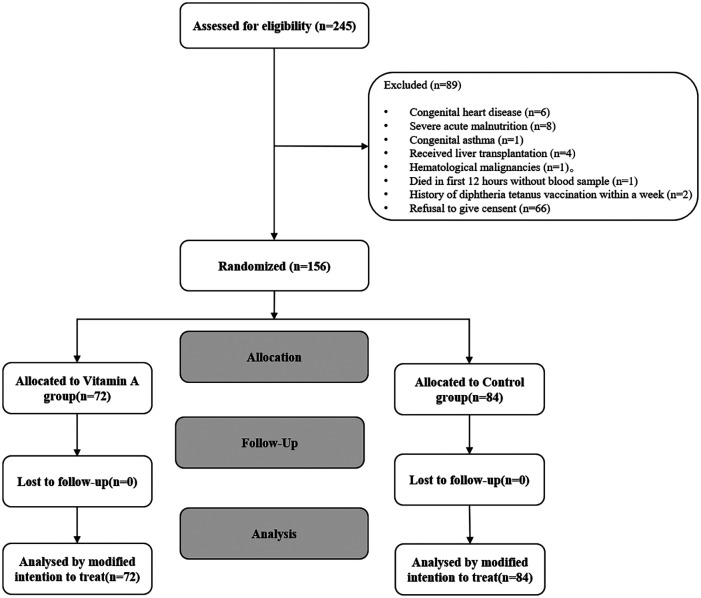
Consort study flow.

From June 2020 to March 2024, a total of 156 children (VAS group, *n* = 72; placebo group, *n* = 84) were enrolled according to the inclusion criteria (Figure 2). The median baseline serum VA level among all enrolled patients was 147.22 ng/ml (IQR: 97.20–258.04), with 65.2% exhibiting VA levels below the normal range. The baseline demographic and clinical characteristics are summarized in Table 2. The mean age of participants was 52 ± 49 months, with males constituting 63% of the total population. Among the enrolled patients, 85.3% were of Han ethnicity, and 89.8% had undergone surgical treatment. Severe sepsis was diagnosed in 63 children, while septic shock was identified in 52 children. The most common primary infection sites were pulmonary (*n* = 63, 40.4%) and multiple infections (*n* = 47, 30.1%). Mean arterial pressure (74.64 ± 14.72 vs. 70.26 ± 14.13 mmHg), heart rate (112.45 ± 27.54 vs. 114.15 ± 26.27 bpm), respiratory rate (26.00 ± 6.25 vs. 25.97 ± 6. 54 breaths/min), and body temperature (37.31 ± 0.85 vs. 37.27 ± 0.95°C) were comparable between the placebo and VA-treated groups at baseline (Table 3). Both groups exhibited similar illness severity according to PRISM III and pSOFA scores.

**Table 2:** Baseline characteristics and clinical characteristics of children with sepsis.

Variable	Control group (*N* = 84)	VA group (*N* = 72)	*P* -value
Age(months)	36 (12, 72)	36 (12, 90)	*P* = 0.833^a^
Sex, *n* (%)			*P* = 0.414^b^
male	56 (67.2%)	42 (58.5%)	
female	28 (32.8%)	30 (41.5%)	
BMI (kg/m^2^)	16.78 ± 3.82	17.06 ± 4.19	*P* = 0.618^c^
Nationality, *n* (%)			*P* = 0.782^b^
Han	73 (86.9%)	60 (83.3%)	
Tibet	4 (4.8%)	6 (8.3%)	
Yi	4 (4.8%)	4 (5.6%)	
Other	3 (3.5%)	2 (2.8%)	
Infection site, *n* (%)			*P*=0.492^b^
multiple	26 (30.5%)	21 (29.2%)	
intra-abdominal infection	12 (14.5%)	21 (29.2%)	
pneumonia	37 (43.5%)	26 (36.0%)	
bloodstream	1 (1.4%)	1 (1.4%)	
skin and soft-tissue	2 (2.9%)	0 (0.0%)	
intracranial infection	4 (4.4%)	3 (4.2%)	
biliary tract	1 (1.4%)	0 (0.0%)	
mediastinum	1 (1.4%)	0 (0.0%)	
Surgery, *n* (%)			*P* = 0.470^b^
Yes	74 (88.1%)	66 (91.7%)	
No	10 (11.9%)	6 (8.3%)	
Emergency operation, *n* (%)	34 (40.5%)	27 (37.5%)	*P* = 0.587^b^
pSOFA score	5 (3.5, 7)	6 (4, 8)	*P* = 0.698^a^
PRISM III	5 (2, 10.5)	5 (2, 9)	*P* = 0.361^a^
VA (ng/ml)	144.50 (97.53, 212.17)	152.07 (98.82, 283.31)	*P* = 0.238^a^
Severe sepsis, *n* (%)	37 (44.0%)	26 (36.1%)	*P* = 0.204^b^
Septic shock, *n* (%)	28 (33.3%)	24 (33.3%)	*P* = 0.820^b^
Duration of antibiotic use, *n* (%)	20 (12, 36.5)	21 (11.5, 40）	*P* = 0.545^a^
Mechanical ventilation time	8.5 (1, 20)	7.5 (1, 19.3)	*P* = 0.477^a^
Vasoactive drugs, *n* (%)	71 (84.6%)	56 (77.8%)	*P* = 0.693^b^
Blood products, *n* (%)	83 (98.8%)	65 (90.3%)	*P* = 0.018^b^
LOS of PICU, day	16.5 (9.3, 28)	14 (7, 27)	*P* = 0.451^a^
LOS of hospital, day	28 (17, 42)	30 (20, 54)	*P* = 0.428^a^
28-day mortality, *n* (%)	11 (13.1%)	4 (5.6%)	*P* = 0.117^b^
Number of hypoglycemic events	0.17 ± 0.70	0.37 ± 1.59	*P* = 0.489^c^
Hypoglycemia, *n* (%)	4 (5.8%)	6 (10.2%)	*P* = 0.188^b^
VA (ng/ml) 3rd day	173.89 (111.80, 234.99)	207.10 (138.82, 279.97)	*P* = 0.085^a^
pSOFA score			
1st day	4 (3, 7.5)	6 (4, 8.5)	*P* = 0.330^a^
3rd day	4 (2, 7)	4 (2, 6)	*P* = 0.649^a^
PRISM III			
1st day	6 (2, 10)	4.5 (0, 7)	*P* = 0.161^a^
3rd day	6 (6, 12.3)	3.5 (0, 8.5)	*P* = 0.205^a^

Data are presented as means (SD), median (quartile 1, quartile 3) or number (percentage). VA, vitamin A; d, day(s), BMI, body mass index; PICU, pediatric intensive care unit.

^a^ Mann–Whitney *U* test.

^b^ Chi-squared test.

^c^ Independent-samples *T* test.

**Table 3:** Basic vital signs of children with sepsis before and after the intervention.

Vital signs	Control group (*N* = 84)	VA group (*N* = 72)	*P* -value
Mean arterial pressure (mmHg)			
0 day	74.64 (±14.72)	70.26 (±14.13)	*P* = 0.612^a^
1st day	77.28 (±14.91)	74.64 (±16.61)	*P* = 0.405^a^
3rd day	78.64 (±14.29)	71.94 (±10.07)	*P* = 0.161^a^
Pulse (cpm)			
0 day	112.45 (±27.54)	114.15 (±26.27)	*P* = 0.987^a^
1st day	110.87 (±22.96)	111.89 (±22.02)	*P* = 0.980^a^
3rd day	111.17 (±20.83)	99.53 (±20.18)	*P* = 0.771^a^
Respiratory rate (cpm)			
0 day	26.00 (±6.25)	25.97 (±6.54)	*P* = 0.408^a^
1st day	25.21 (±6.29)	26.72 (±6.96)	*P* = 0.608^a^
3rd day	23.50 (±5.45)	25.66 (±6.18)	*P* = 0.518^a^
Body temperature (°C)			
0 day	37.31 (±0.85)	37.27 (±0.95)	*P* = 0.430^a^
1st day	37.18 (±0.72)	37.06 (±0.87)	*P* = 0.815^a^
3rd day	37.10 (±0.67)	36.91 (±0.44)	*P* = 0.024^a^

Data are presented as means (SD), median (quartile 1, quartile 3).

^a^ Independent-samples *T* test.

### Primary and secondary outcomes

#### Primary outcome

The median ICU length of stay was slightly shorter by approximately 3 days in the VAS group compared with the placebo group, but the difference was not statistically significant [14 (7–27) days vs. 16.5 (9.3–28) days *P* = 0.451] (Table 2). After adjusting for infection source, surgical status, and baseline PRISM III scores, ICU length of stay remained comparable between groups (adjusted *β* = 0.003, 95% CI: −3.21 to ∼3.25, *P* = 0.983), consistent with the unadjusted analysis (unadjusted *β* = 0.12, 95% CI: −2.98 to ∼3.22, *P* = 0.937). Similarly, no significant difference in mortality risk was found after covariate adjustment (adjusted OR = 1.05, 95% CI: 0.82∼1.34, *P* = 0.701), aligning closely with the unadjusted findings (unadjusted OR = 1.08, 95% CI: 0.85∼1.37, *P* = 0.543). These results indicate robustness after controlling for potential confounding factors.

#### Secondary outcomes

The secondary outcomes are presented in Tables 2, 4. Statistical analyses revealed no significant differences between the two groups regarding hospital length of stay (*P* = 0.428), 28-day mortality (*P* = 0.117), duration of mechanical ventilation (*P* = 0.477), duration of antibiotic use (*P* = 0.545), incidence of hypoglycemia (*P* = 0.188), and utilization of vasoactive drugs (*P* = 0.693). Additionally, laboratory parameters, including blood gas analysis, routine blood examination, liver function, kidney function, and T lymphocyte counts, showed no significant intergroup differences. After adjustment, the reduction in 28-day mortality in the intervention group remained non-significant (adjusted OR = 0.52, 95% CI: 0.15–1.76, *P* = 0.291). Unadjusted: 4 (5.6%) vs. 11 (13.1%), *P* = 0.117).

**Table 4:** Laboratory test results before and after the intervention.

Variable	Control group (*N* = 84)	VA group (*N* = 72)	*P* -value
Blood routine examination			
Hemoglobin (g/dl)			
0 day	101.95 (±18.52)	104.79 (±25.45)	*P* = 0.209^b^
1st day	97.19 (±16.50)	104.93 (±17.12)	*P* = 0.876^b^
3rd day	100.03 (±13.11)	102.70 (±16.52)	*P* = 0.509^b^
7th day	97.31 (±13.02)	99.91 (±16.24)	*P* = 0.230^b^
Platelet (1,000 cells/ul)			
0 day	238.00 (144.00, 343.50)	248.00 (108.00, 384.50)	*P* = 1.000^a^
1st day	216.00 (130.00, 317.00)	204.00 (123.00, 294.50)	*P* = 0.728^a^
3rd day	209.50 (135.00, 315.25)	240.50 (112.50, 394.00)	*P* = 0.558^a^
7 day	189.00 (83.75, 338.75)	243.50 (109.25, 419.00)	*P* = 0.188^a^
White blood cell (1,000 cells/ul)			
0 day	12.43 (7.77, 17.28)	13.69 (7.11, 20.74)	*P* = 0.576^a^
1st day	9.32 (6.99, 13.51)	10.09 (6.79, 15.28)	*P* = 0.607^a^
3rd day	12.43 (7.21, 15.34)	7.42 (5.19, 12.07)	P* = 0.022^a^
7th day	9.16 (5.81, 14.92)	6.42 (4.89, 11.39)	*P* = 0.116^a^
Lymphocyte percent (%)			
0 day	17.85 (8.60, 31.90)	20.70 (12.85, 32.80)	*P* = 0.385^a^
1st day	18.60 (11.40, 26.15)	18.70 (1.05, 29.90)	*P* = 0.746^a^
3rd day	16.10 (11.40, 26.53)	25.15 (13.90, 38.73)	P* = 0.015^a^
7th day	18.45 (11.88, 31.88)	24.60 (14.08, 38.95)	*P* = 0.160^a^
Segmented neutrophil percent (%)			
0 day	76.00 (58.30, 84.90)	70.95 (±12.70)	*P* = 0.651^a^
1st day	72.55 (67.95, 79.95)	67.63 (±13.71)	*P* = 0.338^a^
3rd day	71.30 (55.90, 77.10)	61.61 (48.28, 75.75)	*P* = 0.204^a^
7th day	64.90 (48.30, 75.60)	64.64 (±15.33)	*P* = 0.658^a^
Monocyte percent (%)			
0 day	6.55 (4.48, 9.40)	6.95 (4.70, 8.28)	*P* = 0.688^a^
1st day	7.90 (5.70, 11.75)	7.25 (5.30, 9.65)	*P* = 0.434^a^
3rd day	6.80 (5.20, 10.50)	8.10 (6.95, 13.20)	*P* = 0.037^a^
7th day	8.10 (6.40, 10.60)	8.30 (6.90, 10.35)	*P* = 0.655^a^
Liver function			
Total bilirubin (umol/L)			
0 day	10.30 (5.60, 26.70)	12.05 (7.93, 77.78)	*P* = 0.377^a^
1st day	10.50 (5.50, 34.35)	13.75 (7.20, 72.43)	*P* = 0.420^a^
3rd day	11.50 (6.00, 28.95)	13.90 (9.10, 41.90)	*P* = 0.151^a^
7th day	11.30 (5.48, 37.80)	9.50 (4.10, 61.60)	*P* = 0.887^a^
Direct bilirubin (umol/L)			
0 day	3.65 (2.45, 13.65)	5.70 (2.60, 24.85)	*P* = 0.506^a^
1st day	4.35 (2.50, 11.13)	7.30 (3.28, 33.93)	*P* = 0.140^a^
3rd day	4.30 (2.60, 11.30)	6.10 (3.95, 25.03)	*P* = 0.110^a^
7th day	5.20 (2.20, 15.10)	5.20 (1.18, 36.98)	*P* = 0.658^a^
Total cholic acid (umol/L)			
0 day	4.00 (1.90, 12.70)	4.90 (2.25, 27.80)	*P* = 0.483^a^
1st day	3.10 (1.80, 9.90)	12.80 (2.75, 22.10)	*P* = 0.098^a^
3rd day	4.10 (3.00, 8.70)	4.95 (2.18, 27.10)	*P* = 0.594^a^
7th day	8.10 (2.30, 33.60)	14.20 (4.38, 55.33)	*P* = 0.325^a^
Alanine aminotransferase ALT (IU/L)			
0 day	36.00 (14.00, 102.50)	30.50 (17.00, 166.50)	*P* = 0.737^a^
1st day	34.00 (12.00, 130.50)	51.00 (13.50, 101.00)	*P* = 0.905^a^
3rd day	32.00 (14.00, 95.00)	31.00 (12.00, 82.00)	*P* = 0.978^a^
7th day	45.50 (14.00, 94.50)	45.00 (22.00, 125.00)	*P* = 0.992^a^
Aspartate aminotransferase AST (IU/L)			
0 day	44.00 (26.50, 188.50)	43.50 (28.00, 90.75)	*P* = 0.654^a^
1st day	36.00 (22.50, 151.00)	54.00 (30.00, 78.00)	*P* = 0.590^a^
3rd day	32.00 (24.00, 103.50)	49.00 (32.00, 101.00)	*P* = 0.204^a^
7th day	37.50 (22.50, 76.75)	53.00 (39.00, 120.00)	*P* = 0.069^a^
Albumin (g/L)			
0 day	33.80 (±6.98)	36.35 (±7.00)	*P* = 0.161^b^
1st day	36.82 (±5.47)	37.63 (±5.06)	*P* = 0.741^b^
3rd day	37.16 (±8.90)	39.57 (±6.61)	*P* = 0.432^b^
7th day	36.48 (±6.07)	38.52 (±4.47)	*P* = 0.983^b^
Globulin (g/L)			
0 day	14.70 (11.45, 19.65)	16.95 (12.66, 22.22)	*P* = 0.262^a^
1st day	15.05 (11.45, 19.65)	17.60 (12.40, 22.40)	*P* = 0.377^a^
3rd day	16.40 (12.70, 20.75)	17.75 (14.08, 22.78)	*P* = 0.458^a^
7th day	16.10 (12.90, 20.00)	18.00 (12.45 ,23.93)	*P* = 0.410^a^
Cholesterol (mmol/L)			
0 day	2.28 (1.61, 3.01)	2.66 (1.86, 3.85)	*P* = 0.163^a^
1st day	2.50 (1.78, 2.81)	2.61 (2.08, 3.14)	*P* = 0.124^a^
3rd day	2.71 (1.75, 3.31)	2.79 (2.03, 3.29)	*P* = 0.358^a^
7th day	2.13 (1.65, 3.21)	3.05 (2.25, 3.92)	*P* = 0.036^a^
Renal function			
Creatinine (umol/L)			
0 day	25.00 (16.00, 32.00)	23.00 (16.00, 33.50)	*P* = 0.786^a^
1st day	19.00 (14.00, 30.00)	18.00 (15.00, 27.50)	*P* = 0.751^a^
3rd day	20.00 (16.00, 28.00)	21.50 (15.75, 27.00)	*P* = 1.000^a^
7th day	20.00 (14.00, 24.00)	21.00 (14.00, 25.00)	*P* = 0.731^a^
Urea (umol/L)			
0 day	4.10 (3.00, 7.35)	4.70 (2.60, 6.75)	*P* = 0.804^a^
1st day	4.10 (2.80, 7.80)	4.10 (2.45, 8.50)	*P* = 0.952^a^
3rd day	4.30 (2.80, 8.40)	5.15 (3.68, 6.63)	*P* = 0.560^a^
7th day	4.85 (2.50, 7.35)	3.8092.80, 5.75)	*P* = 0.756^a^
Glomerular filtration rate ml/(min·1.73 m^2^)	211.99 (190.78, 264.02)	215.67 (±54.11)	*P* = 0.786^a^
0 day	221.61 (197.63, 283.95)	229.74 (±56.53)	*P* = 0.946^a^
1st day	234.16 (198.14, 268.79)	227.99 (±66.16)	*P* = 0.922^a^
3rd day	239.52 (204.74, 275.05)	227.21 (±53.28)	*P* = 0.578^a^
7th day			
Inflammation			
Procalcitonin (ng/ml)			
0 day	1.75 (0.33, 16.56)	1.59 (0.38, 9.37)	*P* = 0.964^a^
1st day	1.43 (0.28, 6.86)	0.58 (0.24, 4.28)	*P* = 0.321^a^
3rd day	0.71 (0.17, 4.06)	0.27 (0.13, 0.81)	P* = 0.032^a^
7th day	0.88 (0.36, 4.22)	0.15 (0.07, 0.84)	P* = 0.001^a^
Immunity			
CD3+ (%)			
0 day	62.90 (56.70, 74.80)	64.60 (52.70, 78.85)	*P* = 0.832^a^
3rd day	69.52 (56.18, 78.10)	67.90 (56.32, 80.58)	*P* = 0.719^a^
CD4+ (%)			
0 day	33.60 (25.20, 38.82)	34.90 (28.85, 45.15)	*P* = 0.127^a^
3rd day	35.59 (29.43, 45.83)	37.05 (27.43, 42.82)	*P* = 0.454^a^
CD8+ (%)			
0 day	24.90 (19.30, 31.60)	24.70 (17.05, 31.25)	*P* = 0.439^a^
3rd day	23.65 (18.63, 32.33)	25.25 (20.43, 33.45)	*P* = 0.681^a^

Data are presented as means (SD) or median (quartile 1, quartile 3).

^a^ Mann–Whitney *U* test.

^b^ Chi-squared test.

* *P* < 0.05.

### Lactate levels

**Figure 3:**
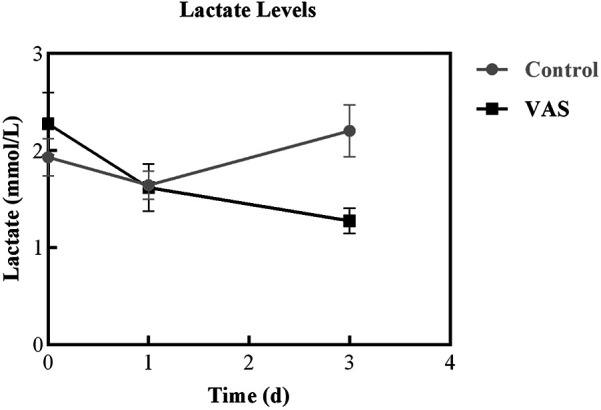
Lactate levels trend.

Baseline lactate levels did not differ significantly between the VAS group (2.28 ± 1.29 mmol/L) and the placebo group (1.93 ± 1.59 mmol/L, *P* = 0.167). However, on the third day post-intervention, lactate levels were significantly lower in the VAS group than in the placebo group (1.28 ± 0.50 mmol/L vs. 2.20 ± 2.21 mmol/L, *P* = 0.001; Table 5). Analysis of lactate trends over time indicated significant differences between the two groups (Figure 3). The VAS group exhibited a marked downward trend (*P* < 0.001), whereas the placebo group demonstrated fluctuating lactate levels (*P* = 0 027). Additionally, the 24-h lactate clearance rate was significantly higher in the VAS group (approximately 25%) than in the placebo group (approximately 10.5%, *P* = 0.036; Table 5).

**Table 5:** Comparison of lactate levels (mmol/L) in two groups at different time.

Variable	Control group(*N* = 84)	VA group (*N* = 72)	*X* ^2^	*P*-value
0 day	1.93 ± 1.59	2.28 ± 1.29	1.91	0.167
1st day	1.67 ± 1.18	1.62 ± 0.92	0.02	0.892
3rd day	2.20 ± 2.21	1.28 ± 0.50	11.55	0.001
*X* ^2^	142.87	373.20		
*P*-value	0.027	<0.001		
Group*time			*X*^2^ = 18.63	*P* < 0.001

### Albumin

**Figure 4:**
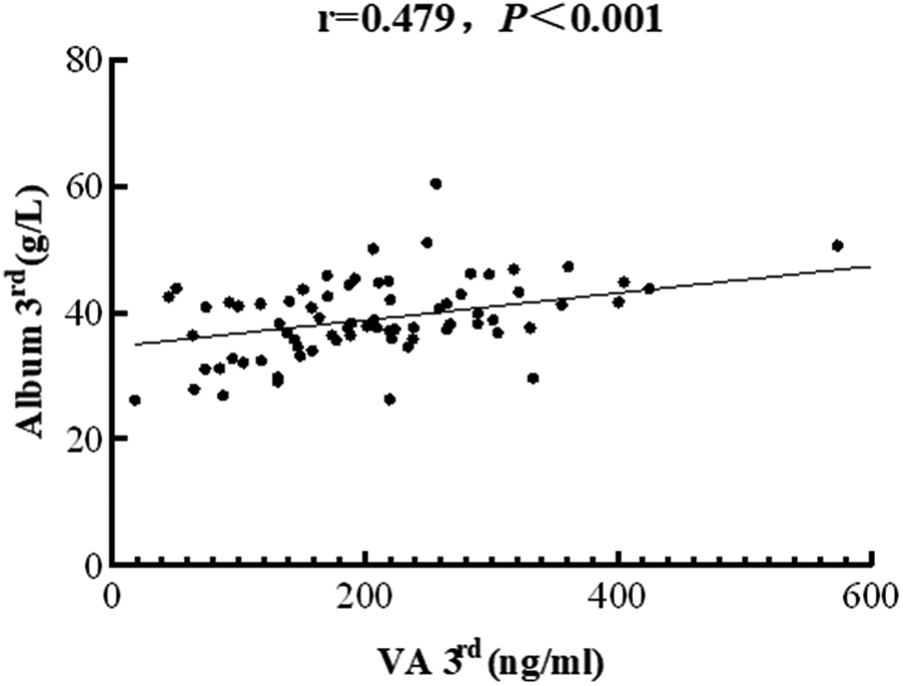
The correlation between serum vitamin A levels and PRISM scores in children with sepsis. The concentrations of vitamin A were negatively associated with albumin levels (correlation coefficient = 0.479, *P <* 0.001).

A serum ALB level below 35 g/L is indicative of hypoalbuminemia, a frequent condition in hospitalized patients, particularly those critically ill (21). Analysis of baseline liver function parameters revealed that hypoalbuminemia was present in 67% of enrolled patients, with no significant difference between the VAS and placebo groups (36.35 ± 7.00 vs. 33.80 ± 6.98 g/L, respectively, *P* = 0.161). On day three after intervention, the proportion of patients with hypoalbuminemia decreased to 44%. Furthermore, a positive correlation between serum VA and ALB levels was observed (*r* = 0.479, *P* < 0.001; Figure 4).

### Inflammatory markers

Typically, PCT levels exceeding 0.5 ng/ml and WBC counts above 9 × 10^3^ cells/µl are indicative of infection (22). In this study, the baseline median PCT level was 4.06 (0.96, 29.58) ng/ml, and the baseline median WBC count was 13.21 (8.04, 19.09) × 10^3^ cells/µl. Notably, patients in the VAS group had significantly lower PCT levels than those in the placebo group on days three [0.27 (0.13, 0.81) ng/ml vs. 0.71 (0.17, 4.06) ng/ml, *P* = 0.032] and seven [0.15 (0.07, 0.84) ng/ml vs. 0.88 (0.36, 4.22) ng/ml, *P* = 0.001] post-intervention (Table 4). Similarly, WBC counts in the VAS group were significantly lower than those in the placebo group on day 3 [7.42 (5.19, 12.07) × 10^3^ cells/µl vs. 12.43 (7.21, 15.34) × 10^3^ cells/µl, *P* = 0.022] and numerically lower, though non-significantly, on day seven [6.42 (4.89, 11.39) × 10^3^ cells/µl vs. 9.16 (5.81, 14.92) × 10^3^ cells/µl, *P* = 0.116] (Table 4). Additionally, the VAS group demonstrated a significantly greater reduction in PCT levels (approximately 73%) within 24 h post-intervention compared with the placebo group (48%, *P* = 0.001).

### Exploratory subgroup analyses

Prespecified subgroup analyses were conducted to evaluate the heterogeneity of treatment effects based on baseline VA status and disease severity.

Stratification by baseline VA status revealed significant differences in outcomes between VA-deficient and VA-sufficient subgroups. In the VA-deficient subgroup (serum VA <200 ng/ml; *n* = 102), VAS significantly reduced ICU length of stay compared to placebo (median 12 day vs. 18 day; *P* = 0.032) and 28-day mortality (3.8% vs. 15.2%; *P* = 0.047). In contrast, the VA-sufficient subgroup (serum VA ≥200 ng/ml; *n* = 54) showed no significant differences regarding ICU length of stay (median 14 vs. 15 day; *P* = 0.52) or mortality (7.1% vs. 10.0%; *P* = 0.67). A significant interaction effect between treatment and subgroup was observed for ICU length of stay (*P* = 0.018 for interaction). After adjusting for covariates, the reduction in ICU length of stay (adjusted median difference: −5.2 days, 95% CI: −9.8 to −0.6, *P* = 0.027) and mortality (adjusted OR = 0.22, 95% CI: 0.05–0.94, *P* = 0.041) remained significant within the VA-deficient subgroup.

**Figure 5:**
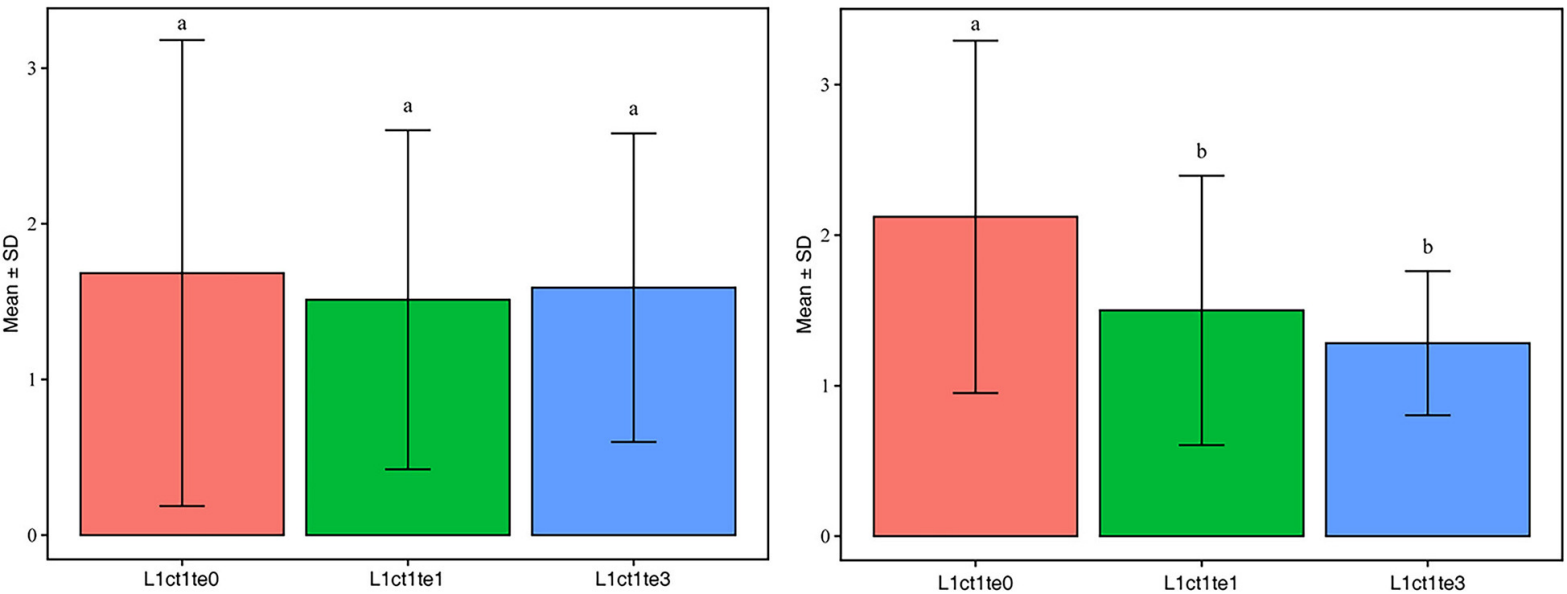
Analysis of variance for lactate. This section of the analysis uses R (completed in 4.4.1), calculates group mean (Mean) and standard deviation (SD) using dplyr package, and performs single factor ANOVA [aov() function] using stats (built-in package). Multiple Tukey HSD comparisons are performed using multcomp package [Tukey HSD() function].

Further stratification by PRISM III scores (median = 10) revealed differential treatment effects. In the high-risk subgroup (PRISM III >10; *n* = 76), patients receiving VAS demonstrated significantly greater lactate reduction (group × time interaction *P* = 0.003 by GEE) despite comparable ICU lengths of stay (median 16 day vs. 18 d; *P* = 0.25). In contrast, no significant treatment benefit was observed in the low-risk subgroup (PRISM III ≤10; *n* = 80) across all evaluated outcomes (all *P* > 0.10), and the interaction between treatment and subgroup was not statistically significant (*P* > 0.05) (Figure 5).

### Severity score

**Figure 6:**
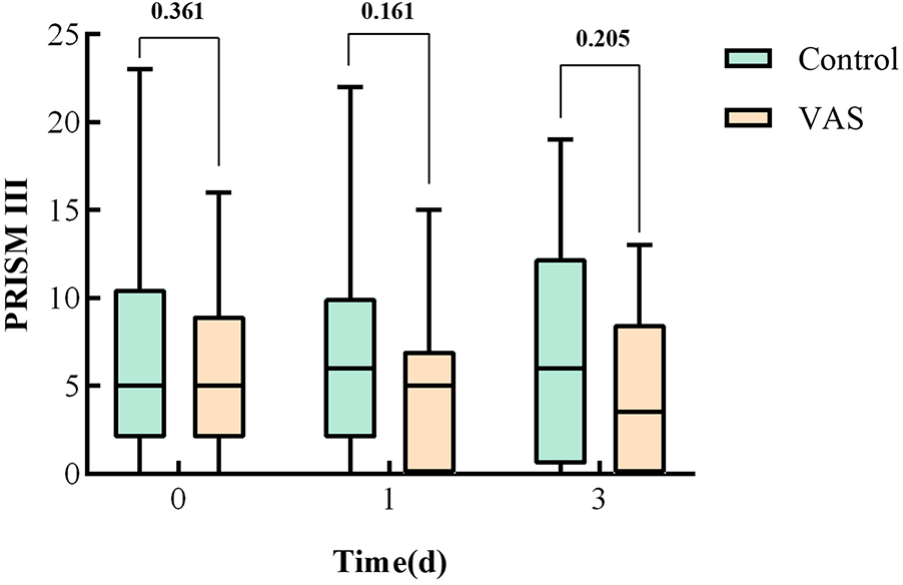
Severity score of two groups in children with sepsis.

Severity scores for the two groups are demonstrated in Table 2. There were no significant differences between groups in baseline pSOFA scores [6(4, 8) in the VAS group vs. 5 (3.5, 7) in the placebo group; *P* = 0.698] or PRISM III scores [5(2, 9) in the VAS group vs. 5 (2, 10.5) in the placebo group; *P* = 0.361]. Dynamic assessments of disease severity revealed that children in the VAS group had numerically lower median PRISM III scores on day 3 after treatment compared to the placebo group, though this difference was not statistically significant [3.5(0, 8.5) vs. 6(6, 12.3); *P* = 0.205] (Figure 6).

#### Adverse events

Among the 156 enrolled patients, 6 required reinsertion after extubation. No patients reported adverse effects related to VAS, such as headache, nausea, vomiting, or diarrhea. None of the adverse events observed were considered causally related to the study intervention.

## Discussion

VA is an essential micronutrient for childhood growth and development, playing a crucial role as an immune modulator. VAD is common in children and can impair immune function, increase infection risk, and lead to excessive inflammatory responses, all contributing to the pathogenesis of sepsis. Previous studies have linked VAD to increased disease severity and impaired immunity in pediatric illnesses such as hand, foot, and mouth disease (9). VAD reduces cellular and humoral immunity and compromises epithelial and mucosal barrier integrity, weakening the body's defenses and increasing susceptibility to infection. Previously, our research group included 160 children with sepsis and 49 healthy controls, finding that VAD was present in 94 (58.8%) subjects with sepsis compared to 6 (12.2%) controls (*P* < 0.001). Hospital and 28-day mortality were higher among children with sepsis and VAD compared to those without VAD, although the differences were not statistically significant. However, VA levels inversely correlated with PRISM scores in children with sepsis and VAD (*r* = −0.260, *P* = 0.012) (11).

Whether VAS can improve prognosis in pediatric sepsis remains unclear, necessitating further research. Thus, we evaluated the effect of adjunctive VA therapy on outcomes in pediatric sepsis by administering exogenous VA. In this study, VA levels in children with sepsis remained significantly lower than normal. However, our prospective data showed no significant differences between the VAS and placebo groups regarding ICU length of stay, 28-day all-cause mortality, or mechanical ventilation duration. Nevertheless, lactate levels in the VAS group exhibited a more significant downward trend within 3 days after intervention. Additionally, the lactate clearance rate within 24 h was significantly higher in the VAS group. Furthermore, PCT and WBC counts were significantly lower in the VAS group compared to placebo. VA levels also appeared positively correlated with serum ALB levels. These results indicate that VAS might regulate lactate levels and improve inflammatory responses in pediatric sepsis.

Exploratory subgroup analyses showed that the benefits of VAS primarily occurred in children with baseline VAD. This subgroup experienced significant reductions in ICU length of stay (*P* = 0.032) and 28-day mortality (*P* = 0.047). The significant interaction (interaction *P* = 0.018) suggests baseline nutritional status may influence treatment responses, aligning with biological plausibility that repletion confers maximal benefit in deficient states. Conversely, the lack of effect in VA-sufficient or low-risk subgroups suggests limited utility for universal supplementation. Future trials should prioritize enrolling VA-deficient pediatric patients with sepsis to validate these findings.

In sepsis, blood lactate levels are widely recognized as markers of disease severity and predictors of mortality. The recent ARISE trial prospectively collected lactate levels from randomly grouped patients. Results indicated that, under similar age and infection conditions, patients with isolated hyperlactatemia had nearly twice the 90-day mortality risk compared to patients with isolated refractory hypotension (23). Masyuk et al. showed that lower 24-h lactate clearance in critically ill patients strongly correlated with adverse outcomes (24). Currently, no clear consensus exists regarding an accurate lactate clearance rate for predicting mortality. However, Vincent suggested that dynamic monitoring of lactate values is necessary, regardless of initial values. The revised Atlanta classification recommends reassessing lactate at 24 h, 48 h, and 7 days after hospital admission. The 24-h time point is particularly important for evaluating treatment efficacy and early disease progression (25). Our study compared the 24-h lactate clearance between groups to evaluate the effect of VA. We found significantly higher lactate clearance in the VAS group compared to placebo (*P* = 0.036). A previous retrospective, single-center cohort study evaluating thiamine in septic shock reported improved lactate clearance (<2 mmol/L) (26). Similarly, our study showed significantly lower lactate levels in the VAS group on the third day after intervention (*P <* 0.01). Furthermore, generalized estimating equation analysis revealed that lactate levels in the VAS group exhibited a more stable downward trend within 3 days post-treatment compared with placebo. Thus, VA may potentially regulate lactate levels in pediatric sepsis. However, baseline lactate levels were not markedly elevated in our study, likely because most participants were perioperative surgical patients with stable vital signs, and many did not meet criteria for septic shock.

ALB is the most abundant protein in human plasma, synthesized by the liver, accounting for approximately 50% of total plasma proteins. It maintains plasma osmotic pressure and binds various nutrients, hormones, and drugs. ALB levels reflect nutritional status and serve as an indicator of liver metabolic function.

In our previous study on VAD in pediatric sepsis, we identified an association between lower VA and decreased ALB levels (11). Data retrospectively collected from four PICUs in China revealed a U-shaped association between serum ALB and mortality among critically ill pediatric patients. Both low and high ALB levels correlated with increased mortality (21). In this study, we similarly observed a positive correlation between VA and serum ALB levels on the third day after intervention.

Basic research has confirmed that ALB interacts with the VA metabolic pathway, playing a crucial role in liver fibrosis development (27). However, the underlying mechanisms remain incompletely understood. Biophysical studies suggest that ALB readily binds RA, possibly at its fatty acid binding site (28, 29). This aligns with a recent study investigating whether proteinuria directly contributes to progressive glomerulosclerosis by suppressing podocyte regeneration. That study indicated that ALB sequesters RA, inhibiting podocyte differentiation (30). Additionally, Lee et al. found that the antifibrotic effects of ALB arise from the downregulation of RA signaling (27). The clinical implications of the positive correlation between ALB and VA may involve liver molecular mechanisms, warranting further long-term foundational research.

An experiment investigating the influence of VAD on immediate and delayed-type hypersensitivity, as well as granulocyte-mediated inflammatory responses in VA-depleted rats, revealed that early sepsis involves excessive inflammation. In the context of VAD, inflammatory responses may become exacerbated, potentially leading to multiorgan dysfunction and increased mortality (31). Similarly, our previous findings indicated that pediatric patients with sepsis and VAD are more susceptible to severe sepsis and septic shock (11). Considering the importance of VA in both anti-inflammatory and proinflammatory immune functions (32), VAD may contribute to exaggerated inflammatory responses in sepsis, resulting in elevated infection markers such as C-reactive protein (CRP) and interleukin-6 (IL-6) (32–35). Mucida and Tanaka et al. demonstrated that VA promotes anti-inflammatory regulatory T-cell differentiation by enhancing Smad3 expression and phosphorylation, as well as forkhead box protein 3 (Foxp3) expression (32, 34). Our clinical trial found elevated PCT levels in children with sepsis. The VAS group had significantly lower PCT levels compared to placebo on the 3rd and 7th days. Yadav et al. indicated that a decrease in PCT levels 24 h post-treatment correlates with a favorable prognosis (36). Our data similarly showed a 73% reduction in PCT levels in the VAS group within 24 h after intervention, significantly greater than that in the placebo group. Additionally, the VAS group had significantly lower WBC counts compared to placebo on the third day, suggesting a positive role for VA in regulating inflammatory responses.

Patients with sepsis frequently exhibit significant lymphocyte depletion, generally attributed to altered immune cell trafficking, potentially contributing to post-sepsis immune suppression (37). Numerous studies confirm that VA and its primary metabolite, RA, regulate T-cell-mediated immune responses. Specifically, they promote regulatory T lymphocyte (Treg) differentiation and maintain T-cell homeostasis. Physiological concentrations of RA also induce IL-2 production, enhancing T-cell proliferation (32). In a prospective trial examining low-dose VAS effects on T-lymphocyte function in neonatal pneumonia, CD4 + and CD8+ T-cell levels increased significantly by the seventh day of treatment in all groups (VA treatment group, conventional anti-inflammatory group, and aerosol inhalation group). However, increases in CD4 + and CD8+ T-cell levels were significantly greater in the experimental group (38). In our trial, CD4 + and CD8+ T-cell levels in the VAS group did not rise significantly by the third day. This result might be due to the predominantly pediatric cohort whose immune systems remain incompletely developed. Therefore, the mechanisms of VA action on lymphocytes in children may differ from those in adults and infants, necessitating further investigation.

### The safety of VA as adjunctive therapy

VA is a micronutrient widely used clinically and generally considered safe. However, acute VA toxicity may result from consuming a single high dose. Common symptoms include increased intracranial pressure, nausea, vomiting, headache, diarrhea, fever, and impaired muscular coordination. Symptoms of chronic toxicity following repeated, prolonged high-dose consumption include vomiting, weight loss, fever, headache, bone abnormalities and pain, enlarged liver, and increased intracranial pressure (39). To ensure safety, we strictly adhered to the World Health Organization's recommended VAS doses for various age groups during our intervention. Throughout the study, no adverse reactions related to the above symptoms were observed among pediatric patients. Additionally, routine blood tests and liver and kidney function assessments showed no abnormalities related to the intervention, supporting the safety of VAS. In our study, we assessed pediatric patients' organ function and disease severity using pSOFA and PRISM III scores. Although differences were not statistically significant, lower PRISM III scores in the VAS group on days 1 and 3 post-intervention indicated a potential reduction in disease severity after VA intervention.

### Clinical implications of mortality trends

Although the reduction in 28-day mortality (13.1% vs. 5.6%, *p* = 0.117) was not statistically significant, the observed absolute risk reduction of 7.5% and relative risk reduction of 57% (RR = 0.43) warrant attention. Such mortality reductions are clinically meaningful in pediatric sepsis trials and align with effect sizes reported in landmark sepsis studies (e.g., activated protein C trial). The lack of statistical significance likely reflects insufficient power due to limited sample size. A *post-hoc* power analysis indicated approximately 300 participants per group would be required to detect this effect (*α* = 0.05, *β* = 0.20).

In the prespecified VA-deficient subgroup (serum VA <200 ng/ml), mortality significantly decreased (15.2% vs. 3.8%, *p* = 0.047). This result suggests that including VA-sufficient patients may have diluted the overall mortality trend. These findings strongly support further exploration in larger trials targeting VA-deficient populations.

Regarding the primary outcome, ICU length of stay may be influenced by non-biological factors (e.g., institutional discharge protocols, availability of rehabilitation beds), potentially limiting its sensitivity in detecting biological effects. Future studies should prioritize patient-centered outcomes, such as mortality or resolution of organ dysfunction.

Significant improvements in biomarkers (e.g., lactate, PCT, WBC count) were observed in the VAS group but did not translate directly into improvements in traditional clinical endpoints, such as mortality or ICU stay. This biomarker-clinical outcome dissociation might arise from several factors. Firstly, although biomarker improvements were statistically significant, their magnitude may have been insufficient to impact definitive outcomes. For example, in patients with mild baseline lactate elevations (median approximately 2 mmol/L), the reduction observed (1.28 vs. 2.20 mmol/L on day 3) may be inadequate to reverse shock pathophysiology. Secondly, a mismatch may exist between the timing of biomarker changes and key prognostic determinants. Early inflammation regulation (PCT/WBC differences on day 3) may primarily affect intermediate pathophysiological processes rather than final recovery trajectories. This hypothesis aligns with findings from the VA-deficient subgroup, which exhibited significant reductions in ICU length of stay (12 vs. 18 days) and 28-day mortality (3.8% vs. 15.2%) alongside substantial biomarker improvements. Furthermore, this study predominantly enrolled patients with moderate disease severity (median PRISM III = 10, 89.8% surgical patients), whose biomarker changes might lack sensitivity in predicting clinical outcomes. In higher-risk sepsis populations, similar biochemical improvements may have clearer clinical relevance. Importantly, biomarker changes themselves carry significant pathophysiological implications: lactate normalization indicates improved cellular oxygen utilization, while reduced inflammatory markers reflect restored immune homeostasis—both critical sepsis treatment targets. Subsequent research should investigate whether these intermediate biomarker effects translate into meaningful long-term patient outcomes, such as functional recovery and organ protection, particularly in VA-deficient populations.

### Limitations

This study has several limitations. First, it was a single-center study with a small sample size drawn from a single clinical setting, potentially limiting generalizability and introducing selection bias. Second, most pediatric patients in our ICU had surgical backgrounds and relatively mild illnesses, leading to lower critical illness scores. Third, the slight imbalance in group sizes (84 controls vs. 72 interventions) might have affected statistical power. Although the total sample size (*N* = 156) exceeded the target (*N* = 144), the non-significant primary outcome (ICU stay) may reflect either genuine equipoise or insufficient power to detect small effects. Notably, the point estimate for ICU stay reduction (14 vs. 16.5 days, *β* = 0.001) aligned directionally with secondary outcomes (e.g., lactate reduction), suggesting clinical relevance that warrants larger trials. Fourth, measurement bias remains possible due to researchers' unblinded assessment of subjective outcomes (pain scores, clinical improvement scales). Fifth, participants were predominantly from Southwest China, mostly Han Chinese (85.3%) and surgical patients (89.8%). This homogeneity may limit applicability to non-surgical populations, rural communities, or diverse ethnic groups, and reduce generalizability to settings with differing medical resources or genetic backgrounds. Future multicenter trials should recruit from diverse healthcare tiers and regions to validate our findings. Sixth, most cases were perioperative (e.g., abdominal or cardiothoracic surgery), differing significantly from medical sepsis (e.g., pneumonia, meningitis) in pathophysiology, surgical stress response, and infection sources. Thus, findings may not extrapolate to non-surgical pediatric sepsis populations. Future studies should stratify enrollment to include comparable proportions of medical and surgical sepsis cases, validating VA's role across heterogeneous etiologies.

## Conclusion

In this study, VA intervention did not significantly affect ICU length of stay or mortality rates in pediatric perioperative surgical patients with sepsis. However, the VAS group demonstrated a more pronounced decrease in lactate levels within 3 days post-intervention and a higher 24-h lactate clearance rate. Additionally, patients receiving VA showed lower inflammation and higher PCT reduction rates within 24 h after treatment. These findings suggest VAS may positively regulate lactate levels and inflammatory responses. The positive correlation between ALB and VA requires further exploration. Moreover, administration of appropriate VA doses in children with sepsis did not lead to adverse reactions, indicating a favorable safety profile for VAS as adjunctive therapy.

## Data Availability

The raw data supporting the conclusions of this article will be made available by the authors, without undue reservation.
